# A rapid and ultra-sensitive dual readout platform for *Klebsiella pneumoniae* detection based on RPA-CRISPR/Cas12a

**DOI:** 10.3389/fcimb.2024.1362513

**Published:** 2024-06-27

**Authors:** Meiying Tan, Lina Liang, Chuan Liao, Zihan Zhou, Shaoping Long, Xueli Yi, Chunfang Wang, Caiheng Wei, Jinyuan Cai, Xuebin Li, Guijiang Wei

**Affiliations:** ^1^ Center for Medical Laboratory Science, Affiliated Hospital of Youjiang Medical University for Nationalities, Guangxi, China; ^2^ Baise Key Laboratory for Research and Development on Clinical Molecular Diagnosis for High-Incidence Diseases, Guangxi, China; ^3^ Key Laboratory of Research on Clinical Molecular Diagnosis for High Incidence Diseases in Western Guangxi, Guangxi, China; ^4^ Department of Clinical Laboratory, Baise People’s Hospital, Guangxi, China; ^5^ School of Food and Chemical Engineering, Liuzhou Institute of Technology, Guangxi, China; ^6^ Department of Neurology, The Affiliated Hospital of Youjiang Medical University for Nationalities, Guangxi, China; ^7^ Modern Industrial College of Biomedicine and Great Health, Youjiang Medical University for Nationalities, Guangxi, China

**Keywords:** *Klebsiella pneumoniae*, recombinase polymerase amplification, CRISPR, Cas12a, lateral flow strip

## Abstract

The bacterium *Klebsiella pneumoniae* (*Kp*) was the primary pathogen of hospital-acquired infection, but the current detection method could not rapidly and conveniently identify *Kp*. Recombinase polymerase amplification (RPA) was a fast and convenient isothermal amplification technology, and the clustered regularly interspaced short palindromic repeats (CRISPR) system could rapidly amplify the signal of RPA and improve its limit of detection (LOD). In this study, we designed three pairs of RPA primers for the rcsA gene of *Kp*, amplified the RPA signal through single-strand DNA reporter cleavage by CRISPR/Cas12a, and finally analyzed the cleavage signal using fluorescence detection (FD) and lateral flow test strips (LFTS). Our results indicated that the RPA-CRISPR/Cas12a platform could specifically identify *Kp* from eleven common clinical pathogens. The LOD of FD and LFTS were 1 fg/μL and 10 fg/μL, respectively. In clinical sample testing, the RPA-CRISPR/Cas12a platform was consistent with the culture method and qPCR method, and its sensitivity and specificity were 100% (16/16) and 100% (9/9), respectively. With the advantages of detection speed, simplicity, and accuracy, the RPA-CRISPR/Cas12a platform was expected to be a convenient tool for the early clinical detection of *Kp*.

## Introduction

The bacterium *Klebsiella pneumoniae* (*Kp)*, belonging to the *Enterobacteriaceae* family (Tominaga, 2018), was a Gram-negative conditional pathogen that commonly colonized various sites such as the oral cavity, skin, respiratory tract, urinary tract, and intestines. *Kp* infection predominantly occurred in people with low immunity (Podschun and Ullmann, 1998; Li et al., 2014), was a main reason for hospital-acquired infection (Pitout et al., 2015), and could result in bacteremia, stroke-associated pneumonia, sepsis, urinary tract infections, liver abscesses, and so on (Wang et al., 1998; Chen et al., 2013; Yao et al., 2015). Moreover, the presence of a severe *Kp* infection often indicated a significant risk of mortality, with reported mortality rates ranging from 16% to 40% (Namikawa et al., 2019). With the use of antimicrobial drugs, *Kp* resistance was increasing and the emergence of multi-drug-resistant *Kp*, especially carbapenem-resistant *Kp*, had made infection with *Kp* a major global public health concern (Zhang et al., 2015). Therefore, the early detection of *Kp* was very important.

Current detection methods for *Kp* mainly include bacterial culture, biochemical identification, immunological assays, and molecular biology technologies. Among these methods, the culture method was considered the gold standard, but it required at least 24 hours of incubation time (Kloss et al., 2013). Some *Enterobacteriaceae* had a high degree of similarity, and identification and differentiation of *Enterobacteriaceae* based on biochemical characterization methods were prone to produce erroneous results (Zamkan and Mohamed, 2018). Matrix-assisted laser desorption ionization time-of-flight mass spectrometry was also commonly used for the identification of *Kp* (Huang et al., 2022) but still required bacterial culture and a long diagnostic time. Molecular diagnostic methods based on nucleic acid detection obviated the need for bacterial culture, thereby being suitable for early clinical diagnosis of *Kp*. The commonly used nucleic acid detection methods for *Kp* included polymerase chain reaction (PCR), isothermal amplification, etc. Compared to traditional detection methods based on bacterial culture, PCR significantly reduced the detection time. However, the dependence of PCR on sophisticated and expensive instruments restricted its application for point-of-care test and in resource-poor areas (Dai et al., 2019), and the detection time of PCR was still longer than that of isothermal amplification. The isothermal amplification method, characterized by its simple instrumentation and rapid reaction time, demonstrated enormous potential to replace PCR.

**Figure 1:**
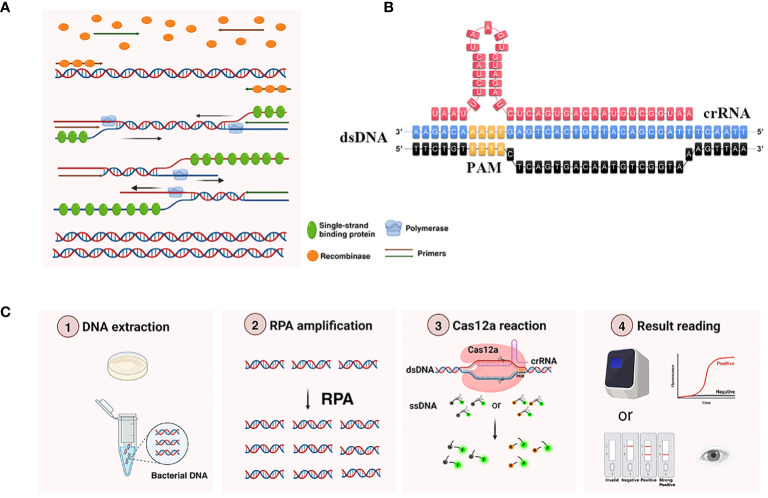
Working principle of RPA-CRISPR/Cas12a platform. **(A)** Principle of RPA amplification. Recombinase bound to the primer to form a complex, and after searching for homologous sequences in the template DNA, the complex inserted into the double-stranded DNA to form a D-loop structure and initiated a strand displacement reaction. At the same time, the template strand that was replaced bound to a single-stranded binding protein to keep the single strand stable. DNA polymerase performed chain extension from the 3’ -OH end of the primer to form a new complementary strand. **(B)** The detailed sequence of recognition site between the crRNA and target DNA. PAM, protospacer adjacent motif, was shown in yellow. Only when crRNA recognized the target DNA, Cas12a could bind the complex of crRNA and target DNA and obtain the ability to trans-cleavage ssDNA reporter. **(C)** Main components and workflow of RPA-CRISPR/Cas12a platform. The extracted DNA was added to the RPA amplification system to obtain more target DNA. Then added the RPA product into CRISPR/Cas12a detection system, when the ssDNA reporter was labeled with FAM at the 5’ end and BHQ1 (or Biotin) at the 3’ end was cleaved by the trans-cleavage ability of Cas12a, the corresponding fluorescence signals could be generated, and the results could be detected by the real-time fluorescence PCR detector (or LFTS).

Recombinase polymerase amplification (RPA), a novel isothermal amplification technology, exhibited distinct characteristics including fast reaction speed, low reaction temperature (37–42°C), and low necessity for sophisticated instrumentation (Dai et al., 2019).Two important enzymes were used in RPA: recombinase and polymerase (Piepenburg et al., 2006). The recombinase could release DNA double strands during replication at low temperature, while polymerase could synthesize new DNA strands (Daher et al., 2016). The principle of RPA was shown in **Figure 1A**. Compared to other isothermal amplification method such as loop-mediated isothermal amplification (LAMP), the important advantages of RPA included its high tolerance to inhibitors in complex samples and easier primer design, whereas the LAMP principle was complex, required multiple pairs of primers, and had a high false positive rate (Dong et al., 2015). Therefore, RPA had attracted a great deal of attention from researchers.

Clustered Regularly Interspaced Short Palindromic Repeats (CRISPR) was a revolutionary gene editing technology first discovered in the immune system of bacteria (Jansen et al., 2002). The CRISPR system demonstrated remarkable attributes of high specificity, sensitivity, and programmability, and was widely employed for nucleic acid detection. Cas12a was an important enzyme of the CRISPR system (Chen et al., 2018). In 2018, Chen et al. reported that CRISPR/Cas12a could precisely cis-cleave target sequences under CRISPR RNA (crRNA) guidance, non-specific trans-cleave ssDNA reporter to amplify the signal of RPA, and successfully used to detect papillomavirus in human patient samples (Chen et al., 2018). The SHERLOCK platform developed by Gootenberg et al. integrated RPA with CRISPR/Cas13a, and was successfully used to detect Zika and Dengue viruses as well as the genotype of human DNA (Gootenberg et al., 2017). Moreover, several studies had reported that pathogenic microorganisms including SARS-CoV-2 could be detected using RPA combined with CRISPR/Cas12a (Lin et al., 2022; Mao et al., 2022; Xiong et al., 2022; Cao et al., 2023). However, there were no reports about using RPA integrated with CRISPR/Cas12a to detect *Kp*.

In this study, three pairs of RPA primers were designed to target the rcsA of *Kp*. By optimizing the RPA and the CRISPR/Cas12a detection system, a platform for *Kp* detection using fluorescence detection (FD) and lateral flow test strips (LFTS) as the signal readout methods were successfully established, and was further compared with culture and qPCR methods. It was expected to address the clinical need for rapidly and accurately detecting *Kp*.

## Materials and methods

### Bacterial strains and DNA extraction

The information on the pathogens applied in this study was listed in **Supplementary Table S1**. Bacterial DNA was extracted according to the instructions of bacterial DNA extraction kit (TIANGEN, Beijing). DNA concentration was measured using a spectrophotometer, and the OD260/280 value of the qualifying sample should be between 1.8 and 2.0. Unused DNA was stored at -20°C. Sputum samples were extracted using the universal genomic DNA extraction kit (TaKaRa, Beijing).

### RPA primer and crRNA design

The nucleotide sequence of *Kp* rcsA gene (GenBank: AY059955.1) was obtained from NCBI, and three pairs of RPA primers (rcsA-F1R1, rcsA-F2R2, rcsA-F3R3) were designed using Primer Premier 5 software (http://www.premierbiosoft.com/primerdesign/) following the guidelines provided by TwistAmp™ Basic Kit (TwistDx, US). The primer pairs were initially verified for specificity using the NCBI online primer design tool “primer-blast”, and then the RPA amplification products were analyzed by 2% agarose gel electrophoresis to select the best primers. After screening the best primers, the corresponding crRNA was designed by the CRISPR online tool (http://www.rgenome.net/cas-designer). The fragment in the rcsA gene that was recognized by crRNA was shown in **Figure 1B**. Based on the cleavage principle of CRISPR/Cas12a, 5’labeled FAM and 3’labeled BHQ1 ssDNA reporter was designed for FD method. And 5’labeled FAM and 3’labeled biotin ssDNA reporter was designed for LFTS method. The corresponding sequences were presented in **Supplementary Table S2**. Sequence synthesis was performed by Nanning GenSys Biotechnology Co., Ltd (Nanning, China).

### RPA amplification system

The total volume of the RPA amplification system was 25 μL, consisting of 14.75 μL of primer-free rehydration buffer, 1.2 μL each of forward and reverse primers (10 μM), 1.25 μL of magnesium acetate (280 mM), 2 μL of DNA template and 4.6 μL water. The preparation of the 25 μL RPA amplification system was conducted as follows: all components without the template DNA and magnesium acetate were thoroughly mixed. The mixture was then added to a reaction tube containing lyophilized enzyme powder, and gently mixed by pipette. The mixture was divided equally into two tubes; 2 μL of template DNA was added into the tubes and 1.25 μL of magnesium acetate was added on the lid of the tube. After centrifugation, the fluorescence signal was detected using a real-time fluorescence PCR instrument at 37°C for 30 min.

### Establishment and optimization of RPA system

The standard strain of *Kp* was used as the positive control and ddH_2_O as the negative control. The RPA reactions were conducted using the designed primer pairs, and the RPA product was analyzed by 2% agarose gel electrophoresis. The best primer pair was selected according to band intensity and specificity. Subsequently, the best primer pair was used to screen out the best RPA reaction temperature among 37°C, 39°C and 41°C. Finally, the RPA amplification time was optimized among 10 min, 15 min, and 20 min under the best primer pair and the best RPA reaction temperature.

### Components of the CRISPR/Cas12a system

After referring to the results reading methods of several detection systems, FD and LFTS were chosen as the results reading methods in this study. The system of FD contained: 38 μL ddH_2_O, 5 μL reaction buffer, 1 μL Cas12a (1 μM), 2 μL crRNA (1 μM), 2 μL ssDNA (10 μM), and 2 μL RPA product. A qPCR instrument (Lightcycler96 qPCR instrument, Roche) was used for fluorescence detection of the FD. The system of LFTS contained: 30 μL ddH_2_O, 5 μL reaction buffer, 1 μL Cas12a (1 μM), 10 μL RPA product, 2 μL crRNA (1 μM), and 2 μL ssDNA (10 μM). The mixture was incubated at 37°C for 30 min and then made up to 80 μL with ddH2O, and finally inserted into the LFTS and the results were read within 10 min. The gray value of the LFTS was scanned by Image J.

### Validation and optimization of the trans-cleavage capability of CRISPR/Cas12a

The trans-cleavage capability of CRISPR/Cas12a was validated by designing eight different reaction systems which were in the absence of one or two components. The trans-cleavage capability of CRISPR/Cas12a was indicated only if the ssDNA reporter was successfully cleaved and fluorescence was detected. In order to increase the performance of the CRISPR/Cas12a detection system, we conducted the optimization for the ssDNA concentrations and the ratios of crRNA/Cas12a. The ssDNA concentrations were set from 100 nM to 600 nM. The ratios of crRNA/Cas12a were set as 1 μL:1 μL, 1 μL:2 μL, 1 μL;3 μL, 2 μL:1 μL, 2 μL;2 μL, 2 μL:3 μL, 3 μL:1 μL, 3 μL:2 μL, 3 μL:3 μL.

### The specificity and limit of detection (LOD) of RPA-CRISPR/Cas12a platform

The bacterial suspensions of all standard strains were made to the same concentration (2.0 McFarland Scale or 6×10^8^CFU/mL) by using the McFarland method, then the DNA of all standard strains was extracted. The RPA-CRISPR/Cas12a platform was used to detect all pathogens in **Supplementary Table S1** to validate the specificity of the platform. Due to limited experimental conditions, it was difficult to obtain actual *M. pneumoniae*, influenza A virus, influenza B virus, and respiratory syncytial virus, so plasmids and lentiviruses were used as the targets, while other bacteria were actual standard strains. Moreover, the DNA concentration of *Kp* was adjusted to 100 pg/μL, 10 pg/μL, 1 pg/μL, 100 fg/μL, 10 fg/μL, and 1 fg/μL. Then these DNA solutions of different concentrations were detected by the RPA-CRISPR/Cas12a platform one by one.

### The performance of RPA-CRISPR/Cas12a platform in clinical sputum samples testing

To assess the clinical application effect, we selected 16 cases of *Kp*-positive samples and 9 cases of *Kp*-negative samples with reference to the results of clinical culture method, and qPCR was used for further identification. The clinical samples collected from 15 cases were then tested using RPA-CRISPR/Cas12a. The qPCR was amplified using rcsA-PCR-F and rcsA-PCR-R primers, and the primer sequences were shown in **Supplementary Table S2**. The qPCR was carried out using a two-step method according to the kit instructions (Hieff qPCR SYBR Green Master Mix, Yeasen Biotechnology), and the reaction program was as follows: pre-denaturation at 95°C for 5 min. 40 cycles with the following parameters: denaturation at 95°C for 10 s, annealing at 60°C for 30 s. The melting curve analysis was set to the default value of the instrument.

### Statistical analysis

Data were recorded using WPS Office software, statistically analyzed using IBM SPSS Statistics 24 software, and GraphPad Prism 9 was used for picture drawing. Fluorescence detection results were compared by two-tailed Student’s t-test. Differences were considered significant at *P* < 0.05.

## Results

### Principle of the RPA-CRISPR/Cas12a detection platform

The principle of the RPA-CRISPR/Cas12a platform for *Kp* detection was shown in **Figure 1C**. Initially, the target DNA was amplified using RPA. Subsequently, the RPA product was introduced into the CRISPR/Cas12a detection system. CRISPR/Cas12a was activated after the formation of the Cas12a-crRNA-target DNA complex, and then precisely cis-cleaved target sequences under crRNA guidance, non-specific trans-cleaved ssDNA reporter to amplify the signal of RPA. The fluorescence generated by ssDNA reporter cleavage could be captured using a real-time quantitative PCR instrument. When the ssDNA reporter ends were labeled with FAM and biotin, respectively, the outcomes could be visualized through LFTS for the point-of-care test.

### Optimization of RPA system

**Figure 2:**
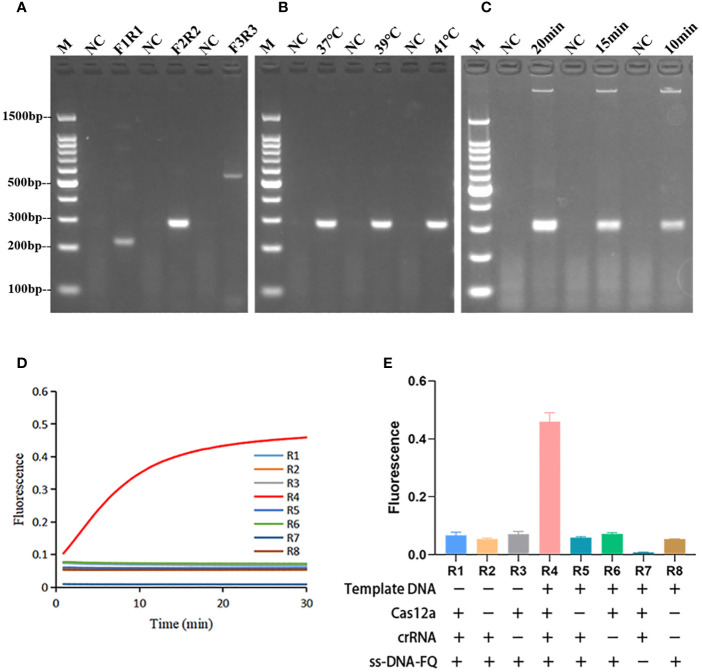
Optimization of RPA reaction conditions and validation of Cas12a cleavage activity. **(A)** RPA primer screening, F3R3 with the highest amplification efficiency was selected as the best primer. **(B)** Optimization of RPA amplification temperature. The RPA amplification temperature was selected to be 37°C. **(C)** Optimization of RPA amplification time. The RPA amplification time was chosen to be 20 min. M, marker. **(D)** Plot of the validated trans-cleavage activity of Cas12a. n=3 replicates. **(E)** Endpoint fluorescence values of Cas12a trans-cleavage activity experiments and information on the components lacking in each reaction. n=3 replicates. R1-R8 represented 8 reactions with different components.

In the primer screening experiments, three pairs of primers were designed. **Figure 2A** showed that the RPA product band amplified by the F2R2 primer pair exhibited the highest intensity and no nonspecific amplification. Therefore, F2R2 was selected as the best primer pair for subsequent experiments. Subsequently, optimization of the RPA amplification temperature was performed. There were no significant differences among the intensities of different bands amplified at 37°C, 39°C, and 41°C (**Figure 2B**), thus 37°C was chosen as the best temperature. Finally, the reaction time of RPA was further explored under the conditions of optimal primer pair and temperature. As demonstrated in **Figure 2C**, the brightest RPA band was produced after amplifying for 20 min. In summary, the best amplification conditions for RPA were: F2R2 primer pair, 37°C, and 20 min.

### Validation and optimization of the trans-cleavage capability of CRISPR/Cas12a

**Figure 3:**
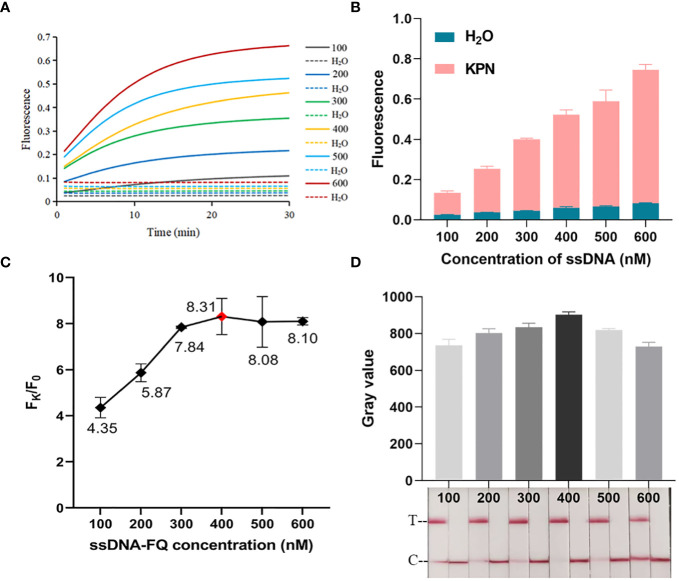
Optimization of ssDNA concentration. **(A)** Real-time fluorescence plot of optimized FD ssDNA concentration. n=3 replicates. **(B)** 30 min endpoint fluorescence map for optimizing ssDNA concentration using FD method. n=3 replicates. **(C)** F_k_/F_0_ in ssDNA concentration optimization. **(D)** Optimization of ssDNA concentration for LFTS. The gray value of the positive band was scanned with ImageJ. The optimal ssDNA concentration was chosen to be 400 nM.

**Figure 4:**
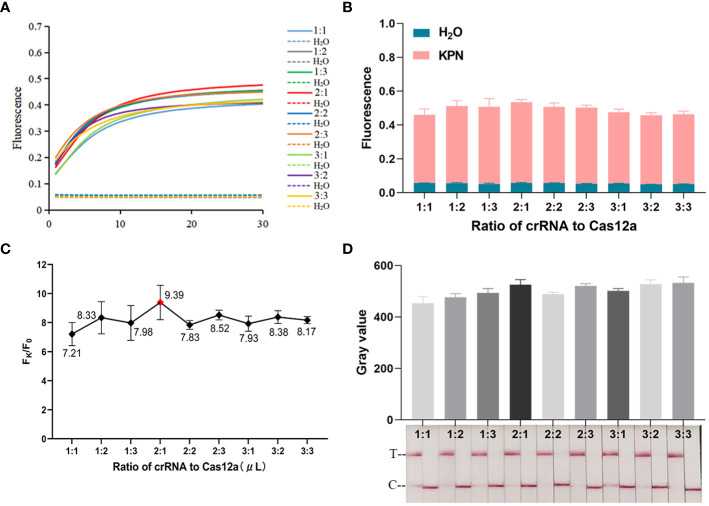
Optimization of crRNA/Cas12a. **(A)** Real-time fluorescence plot of crRNA/Cas12a ratio optimization by the FD method, n=3 replicates. **(B)** 30 min endpoint fluorescence map for optimizing crRNA/Cas12a ratios using FD method, n=3 replicates. **(C)** F_k_/F_0_ in crRNA/Cas12a optimization. **(D)** Optimization of crRNA/Cas12a ratio for LFTS. The gray value of the positive band was scanned with ImageJ. Optimal crRNA/Cas12a ratio selected 2 μL:1 μL.

As shown in **Figures 2D, E**, CRISPR/Cas12a systems without one or two components could not generate fluorescence signal. Fluorescence signal could only be detected when template DNA, Cas12a, crRNA, and ssDNA reporter were present. The ratio of fluorescence intensity of *Kp* (F_k_) to that of ddH_2_O (F_0_) was used to optimize the concentration of ssDNA reporter and the ratio of crRNA/Cas12a. As the concentrations of ssDNA reporter increased from 100 nM to 600 nM, the fluorescence signal also increased gradually. When the ssDNA reporter concentration was 400 nM, F_k_/F_0_ was maximum. Therefore, 400 nM was chosen as the best ssDNA reporter concentration for the FD method (**Figures 3A–C**). When the ratio of crRNA/Cas12a was 2 μL:1 μL, the endpoint fluorescence intensity was strongest and the F_k_/F_0_ ratio was highest (**Figures 4A–C**). In summary, the best cleavage conditions for the reaction system of FD were: 400 nM ssDNA reporter, and the ratio of crRNA/Cas12a was 2 μL:1 μL.

The procedure of optimizing conditions for the LFTS method was similar to that for the FD method. As shown in **Figure 3D**, the strongest band intensity was observed when the ssDNA reporter concentration was 400 nM. As exhibited in **Figure 4D**, when the crRNA/Cas12a were 2 μL:1 μL, 2 μL:3 μL, 3 μL:1 μL,3 μL:2 μL,3 μL:3 μL, the band intensity was strongest. In consideration of reagents cost, the ratio of crRNA/Cas12a was selected as 2 μL:1 μL. In summary, the best cleavage conditions for the reaction system of LFTS were: 400 nM ssDNA reporter, and the crRNA/Cas12a was 2 μL:1 μL.

### The specificity and LOD of RPA-CRISPR/Cas12a platform

**Figure 5:**
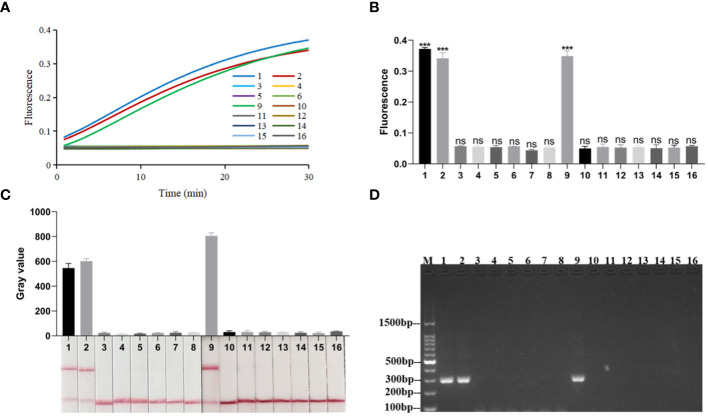
Specificity of the RPA-CRISPR/Cas12a platform. **(A)** Real-time fluorescence profiles of specificity experiments for the FD method, n=3 replicates. **(B)** Plot of the results of the 30-minute endpoint fluorescence values of the specificity experiment for the FD method, n=3 replicates, two-tailed Student’s t-test was used, the results of the assays were compared with negative control, respectively, bars represent mean ± SD, ns, no significance; ***, *P <*0.001. **(C)** Results of specificity validation of LFTS. **(D)** Specificity of the basic RPA. The meanings of the numbers 1–16 in all the pictures were as follows: 1. *Kp* (ATCC1705); 2. *Kp* (ATCC700603); 3. A*.baumannii* (ATCC19609); 4. *Candida albicans* (ATCC10231); 5. *M.pneumoniae*; 6. *Influenza A virus*; 7. *Influenza B virus*; 8. *Respiratory syncytial virus*; 9. *Kp* (ATCC13883); 10. *E.coli* (ATCC25922); 11. *S. typhimurium* (ATCC14028); 12. *S. aureus* (ATCC25923); 13. *E.faecalis* (ATCC35667); 14. *S.pneumoniae* (ATCC49619); 15. *S.aureus*(ATCC29213); 16. negative control; M, marker.

**Figure 6:**
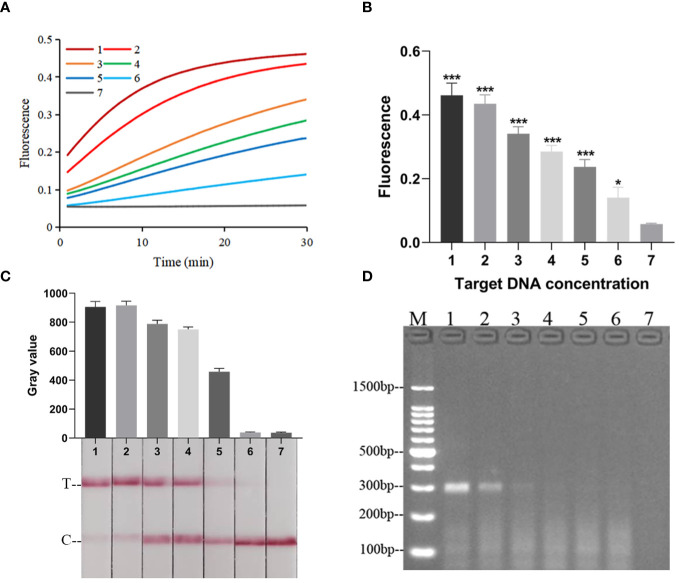
The LOD of the RPA-CRISPR/Cas12a platform. **(A)** Real-time fluorescence profiles of sensitivity experiments for the FD method, n=3 replicates. **(B)** Plot of the results of the 30-minute endpoint fluorescence values of the sensitivity experiment for the FD method, n=3 replicates, two-tailed Student’s t-test was used, the results of the assays were compared with negative control, respectively, bars represent mean ± SD, ns, no significance; *, *p* < 0.05; ***, *P <*0.001. The default was to compare with a negative control. **(C)** Results of sensitivity validation of LFTS. **(D)** Sensitivity of Basic RPA. The meanings of the numbers 1–7 in all the pictures were as follows: 1. 100 pg/μL; 2. 10 pg/μL; 3. 1 pg/μL; 4. 100 fg/μL; 5. 10 fg/μL; 6. 1 fg/μL; 7. negative control; M, marker.

When the RPA-CRISPR/Cas12a platform was used to detect *Kp* and eleven other common clinical pathogens (**Supplementary Table S1**), only for *Kp* was detected as positive by both the FD and LFTS (**Figures 5A–C**). *Kp* was also specifically detected using 2% agarose gel electrophoresis to analyze RPA amplification products (**Figure 5D**). In the LOD validation experiment, FD could detect as low as 1 fg/μL DNA (**Figures 6A, B**), and LFST had a LOD of 10 fg/μL (**Figure 6C**). In contrast, when using 2% agarose gel electrophoresis to directly detect the RPA product, the LOD was only 1 pg/μL (**Figure 6D**). The LOD of the RPA-CRISPR/Cas12a platform was significantly higher than that of the basic RPA.

### The performance of RPA-CRISPR/Cas12a platform in clinical sputum samples testing

**Figure 7:**
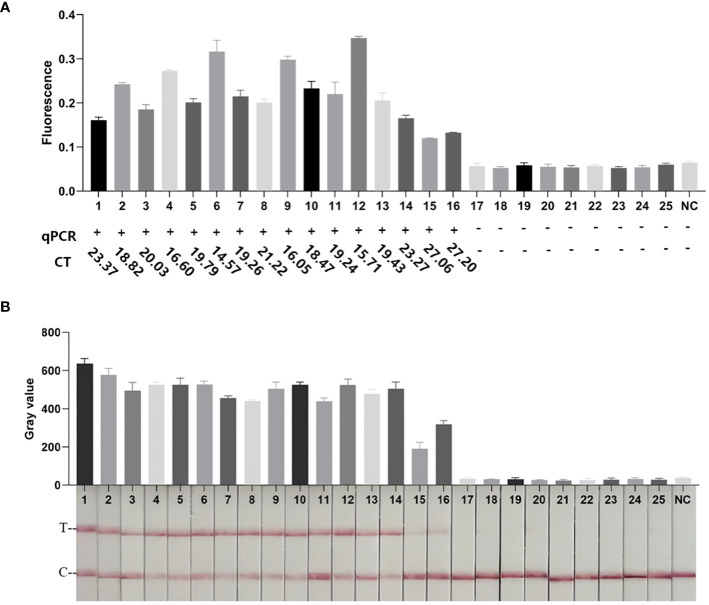
Clinical sample testing. **(A)** The results of FD method and qPCR clinical samples. The endpoint fluorescence value of the FD method at 30 min. The results of qPCR detection and corresponding Ct values. **(B)** Results of clinical samples testing by the LFTS method. All methods had the same results, with samples 1–16 being *Kp*-positive and samples 17–25 being *Kp*-negative.

To evaluate the practicality of the RPA-CRISPR/Cas12a platform, 25 clinical sputum samples were collected. As shown in **Figure 7** and **Supplementary Table S3**, 16 samples were detected as positive by FD, LFTS, culture, and qPCR. 9 samples were detected as negative by FD, LFTS, culture, and qPCR. The RPA-CRISPR/Cas12a platform had comparable detection capabilities to culture methods and qPCR. And it had 100% sensitivity (16/16) and specificity(9/9) compared to gold standard culture method.

## Discussion

*Kp* was a frequently occurring pathogen that was closely associated with human health and hospital-acquired infection (Pitout et al., 2015) It had become a major global public health threat due to its high level of antimicrobial resistance (Wyres and Holt, 2016). Therefore, prevention and control of *Kp* infection was crucial. At present, the commonly used detection methods for *Kp* mainly included culture method, biochemical identification, nucleic acid detection based on PCR, etc (Dong et al., 2015). However, these methods were time-consuming, laborious, complex, and so on, which were not conducive to their application for point-of-care test and in resource-poor areas (Xu et al., 2022). In recent years, isothermal amplification technology such as LAMP had been gradually applied for the detection of *Kp*. As for LAMP, the reaction principle was complex, multiple pairs of primer increased the difficulty of primer design and complexity of the reaction system, and its false positive rate was relatively high (Sun et al., 2021). In contrast, benefiting from the application of recombinase, the principle of RPA was relatively simpler than LAMP. Additionally, RPA only had a pair of primers, which made the reaction system simpler than LAMP. Therefore, RPA was often considered to be a promising isothermal amplification technology. However, the non-specific amplification of basic RPA might produce false positive results, and the LOD of RPA was not low enough to satisfy the demand for ultra-low concentration of nucleic acid detection (Gong et al., 2022). Therefore, RPA signal amplification was necessary to improve its LOD and specificity. The CRISPR system had been extensively used in nucleic acid detection of pathogenic microorganisms. However, the LOD was also not good enough to detect ultra-low concentrations of nucleic acids when the CRISPR system was used alone for detection (Chen et al., 2022). Therefore, the integrated RPA and CRISPR system might compensate for their respective limitations during the detection of *Kp*.

In the present study, we developed an RPA-CRISPR/Cas12a platform to rapidly and accurately detect *Kp* and improved its performance through the optimization of each component. Eventually, the platform was able to detect *Kp* in 50 min at 37°C. The RPA-CRISPR/Cas12a platform had high specificity and did not cross react with other common clinical pathogens, which may be attributed to the specific primers of RPA and the high specificity recognition of RPA products by the crRNA of CRISPR/Cas12a system. The background of clinical sputum samples was complex, and the samples from patients with initial *Kp* infection always had low bacterial content, so the detection method must had an ultra-low LOD. In this study, the FD could detect DNA concentrations as low as 1 fg/μL, while the LOD of the LFTS was 10 fg/μL. The LOD of LFTS was poorer than that of FD, which was consistent with previous studies (Li Y et al., 2022). The basic RPA was only able to detect a DNA concentration of 1 pg/μL. The LOD of the RPA-CRISPR/Cas12a platform was significantly lower than that of the basic RPA, which could be attributed to that the Cas12a enzyme trans-cleaved the ssDNA reporter to exponentially amplify the RPA signal.

Herein, the RPA-CRISPR/Cas12a platform established in this study was further compared with the methods in other studies. The RPA-CRISPR/Cas12a platform was able to detect a lower concentration of *Kp* DNA than the RPA, RAA, LAMP, and LAMP-CRISPR/Cas12a method (Li N et al., 2022; Hou et al., 2022; Poirier et al., 2022; Qiu et al., 2022). In addition, the specificity of the RPA-CRISPR/Cas12a platform was the same as that of RPA (Hou et al., 2022), LAMP (Dong et al., 2015; Liao et al., 2020; Poirier et al., 2022), and LAMP-CRISPR/Cas12a (Qiu et al., 2022), and the sensitivity was the same as that of RPA (Li et al., 2022), RAA (Hou et al., 2022), and LAMP (Dong et al., 2015; Liao et al., 2020; Poirier et al., 2022). The RAA-LFS (Hou et al., 2022) was less specific than the RPA-CRISPR/Cas12a platform (84.6% vs 100%). This might be related to the further identification of the CRISPR/Cas12a, the selection of specific genes, and the stability of the test strips. Additionally, because the LOD of the LAMP-CRISPR/Cas12a was higher than that of the RPA-CRISPR/Cas12a platform, the LAMP-CRISPR/Cas12a (Qiu et al., 2022) was less sensitive than the RPA-CRISPR/Cas12a platform. As for the detection time, RPA, RAA, and LAMP did not combine with signal amplification step like CRISPR/Cas12a, thus their detection time was shorter than the RPA-CRISPR/Cas12a platform (Hou et al., 2022; Li et al., 2022; Poirier et al., 2022). The use of LFTS readout was conducive to point-of-care testing, but it was less stable than fluorescent testing when LFTS strips were exposed to air for a long time, which might more easily produce false-positive results (Hou et al., 2022; Li et al., 2022; Park et al., 2022). Furthermore, it was not suitable to use the LFTS strips to identify the results with the naked eye when the intensity of the detection band was very low (Xiong et al., 2022). As for LAMP, the colorimetric methods such as SYBR Green were usually used to achieve results readout by the naked eye, which was not easy to discern color changes when low-concentration samples were detected. Compared to LFTS and the colorimetric methods, the instrumental readout of the fluorescence signal was more stable and accurate. In our study, the FD used a real-time fluorescent quantitative PCR instrument to detect fluorescence signal and was suitable to be applied in the laboratories of resourceful areas. In contrast, the LFTS method used LFTS to detect fluorescence signal and was suitable to be applied for point-of-care testing and in the laboratories of resource-poor areas.

There were some limits in the present study. Firstly, although the RPA and CRISPR/Cas12a reactions could be completed within 50 min, the commonly used DNA extraction from clinical samples by commercial kits was always time-consuming. A fast, convenient, and efficient DNA extraction method for sputum samples needed to be developed in the future. Secondly, although the RPA-CRISPR/Cas12a platform had demonstrated 100% sensitivity and 100% specificity in clinical sputum samples testing, the sample size in our study was still relatively small, and a larger number of clinical samples were still required to further confirm the performance of this platform in the future.

In summary, we developed a novel detection platform for *Kp* based on the RPA-CRISPR/Cas12a. The platform was capable of detecting *Kp* quickly, easily, and accurately at room temperature. The platform had detection capabilities comparable to culture and qPCR methods. And it was easy to operate without relying on precise and complex temperature control equipment. It could be used for rapid and accurate clinical diagnosis of *Kp* in the future, especially in resource-poor areas.

## Data availability statement

The original contributions presented in the study are included in the article/**Supplementary Material**. Further inquiries can be directed to the corresponding authors.

## Ethics statement

The studies involving humans were approved by Medical Ethics Committee of the Affiliated Hospital of Youjiang Medical University for Nationalities. The studies were conducted in accordance with the local legislation and institutional requirements. Written informed consent for participation was not required from the participants or the participants’ legal guardians/next of kin because Written informed consent for participation was not required for this study in accordance with the national legislation and the institutional requirements. Written informed consent was not obtained from the individual(s) for the publication of any potentially identifiable images or data included in this article because Written informed consent for participation was not required for this study in accordance with the national legislation and the institutional requirements.

## Author contributions

GW: Writing – review & editing. MT: Data curation, Methodology, Validation, Writing – original draft, Writing – review & editing. LL: Writing – original draft. CL: Writing – review & editing. ZZ: Writing – review & editing. SL: Writing – review & editing. XY: Writing – review & editing. CWa: Writing – review & editing. CWe: Writing – review & editing. JC: Writing – review & editing. XL: Writing – review & editing.
