# Supplementary material for: A rapid and ultra-sensitive dual readout platform for Klebsiella pneumoniae detection based on RPA-CRISPR/Cas12a
Source: Front Cell Infect Microbiol. 2024 Jun 27;14:1362513. doi: 10.3389/fcimb.2024.1362513 (PMC11236598; doi:10.3389/fcimb.2024.1362513)
Supplement: Supplementary file 1 [file Table_1.docx]

**Table S1** Information on pathogenic microorganisms used in this study

| **Name** | **Category number** | **Source** |
| --- | --- | --- |
| *K. pneumoniae* | ATCC13883 | American Type Culture Collection |
| *K. pneumoniae* | ATCC1705 | American Type Culture Collection |
| *K. pneumoniae* | ATCC700603 | American Type Culture Collection |
| *E.coli* | ATCC25922 | American Type Culture Collection |
| *S.typhimurium* | ATCC14028 | American Type Culture Collection |
| *S.aureus* | ATCC25923 | American Type Culture Collection |
| *E.faecalis* | ATCC35667 | American Type Culture Collection |
| *S.pneumoniae* | ATCC49619 | American Type Culture Collection |
| *S.aureus* | ATCC29213 | American Type Culture Collection |
| *A.baumannii* | ATCC19609 | American Type Culture Collection |
| *Candida albicans* | ATCC10231 | American Type Culture Collection |
| *M.pneumoniae* | 240122-2 | Sansure Biotech Inc |
| *Influenza A virus* | 240122-2 | Sansure Biotech Inc |
| *Influenza B virus* | 240122-2 | Sansure Biotech Inc |
| *Respiratory syncytial virus* | 240122-2 | Sansure Biotech Inc |

**Table S2** Nucleic acid sequences used in this study

| **Name** | **Sequence (5’- 3’)** | **Primer size (nt)** | **Amplicon size (bp)** |
| --- | --- | --- | --- |
| rcsA-F1 | GCTATTTGCGGGTACGGAAGAATCTGCTAA | 30 | 220 |
| rcsA-R1 | GCTTTGATGTTCATTTGCGTTGAGATTTGC | 30 |  |
| rcsA-F2 | ACCCGGCGACGCTGTTTGTTATCTTTATGT | 30 | 286 |
| rcsA-R2 | GATACCGTCTTCGCTTTGATGTTCATTTGC | 30 |  |
| rcsA-F3 | TGTGCAGCTATACCCGGTTGGGATTGACGG | 31 | 552 |
| rcsA-R3 | CGATATGATAAATCACCTGCTTATTATGCGTTTG | 34 |  |
| crRNA | UAAUUUCUACUAAGUGUAGAUCUCAGUGACAAUGUCGGUAA | 41 |  |
| FQ | FAM-TTATT-BHQ1 | 5 |  |
| FB | FAM-TTATT-Biotin | 5 |  |
| rcsA-PCR-F | TTGTTATCTTTATGTCGCTGG | 21 |  |
| rcsA-PCR-R | ATCAAGGTCTTTTGGGGTTA | 20 |  |

**Table S3** Comparison of clinical sample detection results between RPA-CRISPR/Cas12a platform and culture and qPCR method

|  |  | | RPA-CRISPR/Cas12a platform | |  |
| --- | --- | --- | --- | --- | --- |
|  |  | | ＋ | － | Total |
| Culture method | ＋ | 16 | | 0 | 16 |
|  | － | 0 | | 9 | 9 |
|  | Total | 16 | | 9 | 25 |
| qPCR | ＋ | 16 | | 0 | 16 |
|  | － | 0 | | 9 | 9 |
|  | Total | 16 | | 9 | 25 |
